# Dyadic examination of rumination and depressive symptoms in Chinese heterogeneous young couples: the differential role of gender

**DOI:** 10.3389/fpsyt.2024.1447040

**Published:** 2025-01-21

**Authors:** Xiaoyu Liu, Anji Zhou, Zhuyun Jin, Zhuo Rachel Han, Jing Qian, Hui Wang

**Affiliations:** ^1^ Institute of Basic Research in Clinical Medicine, China Academy of Chinese Medical Sciences, Beijing, China; ^2^ Beijing Key Laboratory of Applied Experimental Psychology, Faculty of Psychology, National Demonstration Center for Experimental Psychology Education, National Virtual Simulation Center for Experimental Psychology Education, Beijing Normal University, Beijing, China; ^3^ Business School, Beijing Normal University, Beijing, China; ^4^ Department of Psychology, Faculty of Arts and Sciences, Beijing Normal University at Zhuhai, Zhuhai, China

**Keywords:** gender, rumination, depressive symptoms, romantic relationship, young couples

## Abstract

**Introduction:**

The relationship between rumination and depressive symptoms has been extensively studied over the past two decades. However, few studies have explored how rumination contributes to depressive symptoms within the context of heterogeneous romantic relationships, particularly regarding potential gender differences in these effects. The present study aims to investigate whether rumination is related to four key factors of depressive symptoms (i.e., depressed affect, positive affect, somatic and retarded activity, interpersonal distress) both on the intrapersonal and interpersonal levels among young couples.

**Methods:**

Participants were 148 Chinese young couples (*N* = 296; males: *M* age = 21.94 years, *SD* = 2.40 years; females: *M* age = 21.62 years, *SD* = 2.26 years). Couples completed self-reported questionnaires assessing rumination and depressive symptoms separately, using the Ruminative Response Scale (RRS) and the Center for Epidemiologic Studies Depression Scale (CES-D).

**Results:**

The results of a series of actor-partner interdependence models (APIM) showed that, on the intrapersonal level, rumination was positively and significantly associated with an individual’s own depressed affect, somatic and retarded activity, and interpersonal distress. On the interpersonal level, higher levels of rumination in males were associated with increased depressed affect and interpersonal distress in their female partners. However, no such partner effect was observed for male partners of ruminative females.

**Conclusions:**

These findings suggest that females in romantic relationships, as compared to males, may be more susceptible to the influence of their male partners’ rumination. This study is among the firsts to demonstrate the gender-specific effect in the relationship between rumination and depressive symptoms in young couples.

## 1 Introduction

In recent years, research on the association between rumination and depressive symptoms has expanded significantly (1–3), largely due to the critical role rumination plays in understanding mental health outcomes, particularly depression (4–7). While many studies have examined the relationship between rumination and depressive symptoms at the individual (intrapersonal) level (1, 2), few have adopted a comprehensive perspective or explored these associations within romantic relationships. Moreover, whether rumination affects depressive symptoms differently based on gender within heterosexual romantic relationships remains uncertain. This study aims to enhance our understanding of rumination by examining its influence on both an individual’s own depressive symptoms and those of their partner in young heterosexual couples, as well as investigating potential gender differences in these relationships.

### 1.1 Rumination and depressive symptoms

According to response styles theory (8), rumination is defined as a response style involving a repeated focus on self-related negative feelings during times of distress. Individuals with a ruminative response style dwell on thoughts and behaviors related to the potential causes, meanings, and consequences of their distressed feelings (2, 6). Extensive evidence links rumination with various adverse outcomes, including negative thinking (9), anxiety (10), and post-traumatic stress symptoms (11).

In addition to these negative impacts, rumination has been strongly and consistently associated with depressive symptoms (1, 2). Radloff (12) proposed four major aspects of depressive symptoms: (1) depressed affect, including feelings of worthlessness, helplessness, and hopelessness; (2) somatic and retarded activity, such as loss of appetite and sleep disturbances; (3) absence of positive affect, including diminished feelings of hope, happiness, or enjoyment; and (4) interpersonal distress, characterized by feelings of being unfriendly and disliked. Increased rumination has been found to be associated with each of these aspects of depressive symptoms, including greater depressed affect (1) and higher somatic and retarded activity, such as a reduced ability and motivation to develop effective plans and behaviors for managing problems (13, 14). Regarding interpersonal distress, adolescents and adults who are depressed tend to ruminate more, particularly on stressors involving interpersonal issues (15, 16). However, findings on the association between rumination and positive affect have been mixed. Some studies indicate that engaging in rumination reduces positive affect (17, 18), while others report no significant correlation between rumination and positive affect (19). Therefore, further studies are recommended to clarify the relationships between rumination and different aspects of depressive symptoms, especially the absence of positive affect.

### 1.2 The role of gender

Gender differences in rumination are widely noted, with researchers suggesting that females are more likely than males to ruminate when feeling sad or depressed (20–22). Several explanations have been proposed for why females may be more prone to rumination. First, socialization processes may encourage females to be more emotionally expressive and introspective, leading them to focus more intensely on their emotional experiences, which can promote ruminative thinking (23). For example, Zimmermann and Iwanski (24) argued that females are often socialized to be more attuned to their emotions, and this heightened focus on internal experiences can increase the likelihood of rumination, particularly during emotional distress. Additionally, females may find it more challenging to alleviate negative emotions, making them more susceptible to rumination as they work to process and regulate these feelings (7). Another explanation is females’ tendency to feel responsible for the emotional climate of their relationships, which increases their sensitivity to partners’ behaviors and comments (25, 26). This heightened relational awareness can make females more vigilant in detecting potential interpersonal conflicts or negative cues, potentially leading to rumination. Given the gender disparity in rumination, it is important to understand whether rumination is equally related to depressive symptoms for both males and females.

### 1.3 Rumination in romantic relationship

Recent studies have increasingly focused on rumination and its detrimental effects within romantic relationships (5, 27). For instance, Verhallen et al. (28) examined depressive symptom trajectories following relationship breakups and assessed the impact of rumination impact on these trajectories. They found that individuals with higher levels of rumination experienced more severe depressive symptoms post-breakup, underscoring how rumination amplifies distress in romantic contexts. Additionally, Horn et al. (27) studied couples transitioning to retirement and explored rumination’s impact on their daily adjustment. They observed that on days with elevated rumination, couples reported greater communication difficulties, particularly in understanding the retiree’s disclosures, and experienced more negative emotions. While these studies provide valuable insights, they are limited to specific adult samples, such as couples post-breakup or retirees, and thus leave a gap in understanding how rumination impacts couples in more general populations.

In the limited studies that have examined rumination patterns among general couples, findings consistently indicate that females are more likely than males to engage in rumination (29, 30). For example, Bastin et al. (29) found that adolescent females engaged in co-rumination more frequently than males, which was associated with higher levels of depressive symptoms. This tendency may partly stem from females’ enhanced social perspective-taking abilities, a skill that supports relationship quality but can also lead to empathetic distress in emotionally charged situations (31). Focusing specifically on young couples, the impact of rumination on depression becomes particularly important due to the central role that romantic relationships play in their psychological development. Romantic relationships during early adulthood are characterized by emotional intensity and identity formation, and difficulties within these relationships can significantly affect mental health (32). For instance, young couples may experience heightened emotional reactivity to their partner’s ruminative thoughts, which could exacerbate depressive symptoms. Research indicates that higher levels of rumination not only affect individuals directly but can also contribute to stress and emotional strain within the relationship (28).

While studies have shown that women are more likely to co-ruminate, limited empirical research has directly examined how a partner’s rumination affects both their own and their partner’s depressive symptoms. Moreover, little is known about whether these relationships differ according to the gender of the couples involved. Given the significant role of romantic relationships in the mental health of young adults (28, 32), more research is needed to explore the association between rumination and depressive symptoms among young couples and to determine whether gender differences exist in these relationships.

### 1.4 Advantages of dyadic analysis

To explore the impact of individuals’ rumination on both their own (i.e., intrapersonal) and their partner’s (i.e., interpersonal) depressive symptoms, we employed the actor-partner interdependence model (APIM). The APIM is particularly advantageous for analyzing dyadic data, as it treats the dyad as the unit of analysis, thus accounting for the non-independence of data that naturally arises in close relationships (33). By examining both the “actor effect” (the impact of an individual’s rumination on their own depressive symptoms) and the “partner effect” (the impact of an individual’s rumination on their partner’s depressive symptoms), the APIM enables us to distinguish between intrapersonal and interpersonal influences within the dyadic context, making it well-suited for understanding mutual influences within couples. Due to this advantage, the APIM has become a popular approach in studies of dyadic interactions (34). In the context of our study, using the APIM allows us to examine how one partner’s rumination might resonate within the relationship, potentially impacting the mental health of both partners. This approach supports a comprehensive analysis of the reciprocal influences in couple dynamics, offering valuable insights into how psychological processes like rumination function within intimate relationships.

### 1.5 Current study

Our study is one of the firsts to explore the relationship between rumination and depressive symptoms at both interpersonal and interpersonal levels in Chinese young couples. Specifically, we aim to investigate how an individual’s rumination is associated with their own and their partner’s various dimensions of depressive symptoms (i.e., depressed affect, positive affect, somatic and retarded activity, and interpersonal distress) and to explore whether there were gender differences in these relationships. Based on theoretical considerations, we hypothesized that rumination would be positively associated with both an individual’s own and their partner’s depressive symptoms across all four dimensions. Moreover, we proposed that females might be more susceptible to the influence of their male partner’s ruminative thoughts in relation to their own depressive symptoms.

## 2 Method

### 2.1 Participants

Data were drawn from a study on romantic relationships and psychological well-being among young heterosexual couples. Participants were recruited via flyers posted in the local community. Eligibility criteria required that participants be in a romantic relationship for at least three months, have no clinical diagnosis of psychological disorders (e.g., depression), and not be taking any medication related to mental health. A total of 163 heterosexual couples (*N* = 326) initially agreed to participate. However, nine couples were excluded due to incomplete assessments, and three additional couples were excluded because of technical issues. This resulted in a final sample of 151 couples (*N* = 302). Male participants ranged in age from 17 to 33 years (*M* age = 21.94 years, *SD* = 2.40 years), while female participants ranged from 18 to 27 years (*M* age = 21.62 years, *SD* = 2.26 years). The couples had been in their romantic relationship for an average of 25.64 months (*SD* = 21.19 months, range = 3-96 months).

### 2.2 Procedure

All study procedures were approved by the university’s Institutional Review Board (IRB). Upon arrival at our laboratory, all participants provided written informed consent. They then completed a series of questionnaires, including demographic information, rumination, and depressive symptoms. The entire procedure took approximately one hour. Each couple received 150 RMB (approximately 20 USD) as compensation for their participation.

### 2.3 Measures

#### 2.3.1 Rumination

The ruminative response style in couples was assessed using the Ruminative Response Scale (RRS; 35). The RRS comprises 22 items that assess an individual’s tendency to ruminate in response to sad moods. Participants rate the frequency of their use of ruminative strategies on a 4-point Likert scale (1 = almost never to 4 = almost always), with higher scores indicating a stronger ruminative response style. The original RRS demonstrated good internal consistency (35), and the Chinese version has also showed strong reliability and validity (36). In this study, the RRS exhibited excellent internal consistency, with Cronbach’s α = .920 for males and α = .925 for females.

#### 2.3.2 Depressive symptoms

Couples’ depressive symptoms were measured using the Center for Epidemiologic Studies Depression Scale (CES-D; 12). The CES-D consists of 20 items and includes four subscales: depressed affect (e.g., “I felt that I could not shake off the blues even with help from my family or friends”), positive affect (e.g., “I felt that I was just as good as other people”), somatic and retarded activity (e.g., “I did not feel like eating; my appetite was poor”), and interpersonal distress (e.g., “People were unfriendly”). The Chinese version of the CES-D has shown good psychometric properties (36). In the current study, we calculated internal consistency reliability separately for males and females. The reliability for each CES-D subscale was acceptable: Cronbach’s α for the depressed affect subscale was.827 for males and.874 for females; for the positive affect subscale,.696 for males and.732 for females; for the somatic and retarded activity subscale,.687 for males and.721 for females; and for the interpersonal distress subscale,.640 for males and.671 for females.

### 2.4 Data analysis

In the preliminary analyses, we conducted descriptive statistics and Pearson correlation analyses for the study variables. In dyadic data analysis, non-independence refers to the interdependence of variables within a couple, where one partner’s characteristics or behaviors may affect the other’s (33). Recognizing this interdependence is essential, as it supports treating the dyad as a single unit in statistical models like APIM. To confirm non-independence in our study, we examined correlations between males and females on the same variables, specifically rumination and the four dimensions of depressive symptoms. Significant correlations would indicate non-independence, justifying the use of APIM for further analysis.

Subsequently, we conducted four APIM models to investigate the effects of males’ and females’ rumination on both their own and their partner’s depressed affect, positive affect, somatic and retarded activity, and interpersonal distress, using Analysis of Moment Structures (Amos) version 20.0 software. Given that participant age and relationship duration could influence the observed relationships, we included these variables as covariates in the models. We analyzed the dyad as a unit and used a 95% confidence interval (CI) based on 5,000 bootstrap samples to assess the significance of actor effects (the influence of an individual’s rumination on their own depressive symptoms) and partner effects (the influence of an individual’s rumination on their partner’s depressive symptoms). Unstandardized coefficients were reported in accordance with recommendations from Kenny et al. (33).

## 3 Results

### 3.1 Preliminary analyses

The descriptive statistics and Pearson correlations are presented in **Table 1**. Both females’ and males’ rumination were significantly and positively associated with their own depressed affect, somatic and retarded activity, and interpersonal distress (*p*-values <.05). Additionally, females’ depressed affect was positively correlated with males’ depressed affect (*r* = .306, *p* <.001), and females’ somatic and retarded activity was positively correlated with males’ somatic and retarded activity (*r* = .165, *p* = .043). There was also a positive correlation between females’ and males’ rumination (*r* = .250, *p* = .002). These results suggest a non-independence of study variables within couples.

**Table 1:** Descriptive statistics and bivariate correlations among study variable.

Variable	*M*	*SD*	1	2	3	4	5	6	7	8	9	10	11	12
1. F-depressed	11.14	3.97	—											
2. F-positive	7.58	3.05	-.276^**^	—										
3. F-somatic	8.61	2.77	.687^**^	.206^*^	—									
4. F-interpersonal	2.50	0.91	.588^**^	.185^*^	.497^**^	—								
5. M-depressed	10.19	3.17	.306^**^	-.003	.265^**^	.163^*^	—							
6. M-positive	7.56	3.17	.014	.132	.013	.034	.213^**^	—						
7. M-somatic	8.20	2.54	.202^*^	-.074	.165^*^	.180^*^	.732^**^	.197^*^	—					
8. M-interpersonal	2.32	0.79	.102	-.005	.108	.066	.627^**^	.212^**^	.567^**^	—				
9. F-Rumination	38.83	10.29	.559^**^	.019	.453^**^	.380^**^	.242^**^	-.071	.178^*^	.089	—			
10. M-Rumination	36.99	10.81	.352^**^	.025	.252^**^	.335^**^	.478^**^	.016	.364^**^	.311^**^	.250^**^	—		
11. F-Age	21.62	2.26	-.132	.093	-.161^*^	-.033	-.138	.066	-.104	-.061	-.220^**^	-.058	—	
12. M-Age	22.27	2.50	-.145	.054	-.154	-.098	-.107	.082	-.065	-.024	-.193^*^	-.056	.792^**^	—
13. Length	25.64	21.19	-.168^*^	.014	-.201^*^	-.195^*^	-.221^**^	-.117	-.267^**^	-.261^**^	-.057	-.129	.213^**^	.130

*N* = 151; F = female, M = male; depressed = depressed affect, positive = positive affect, somatic = somatic and retarded activity, interpersonal = interpersonal distress, length = relationship duration in months.

*p <.05, **p <.01.

Moreover, females’ rumination was significantly and positively associated with males’ depressed affect (*r* = .242, *p* = .003) and somatic and retarded activity (*r* = .178, *p* = .029). Similarly, males’ rumination was significantly and positively correlated with females’ depressed affect (*r* = .352, *p* <.001), somatic and retarded activity (*r* = .252, *p* = .002), and interpersonal distress (*r* = .335, *p* <.001).

Finally, a series of paired-sample *t*-tests were conducted to assess potential gender differences in the key study variables. The results indicated a significant difference in depressed affect between females and males (*t* (150) = 2.733, *p* = .007). However, there were no significant gender differences in rumination (*t* (150) = 1.744, *p* = .083), positive affect (*t* (150) = 0.040, *p* = .968), somatic and retarded activity (*t* (150) = 1.470, *p* = .144), or interpersonal distress (*t* (150) = 1.883, *p* = .062).

### 3.2 APIM models

**Figure 1:**
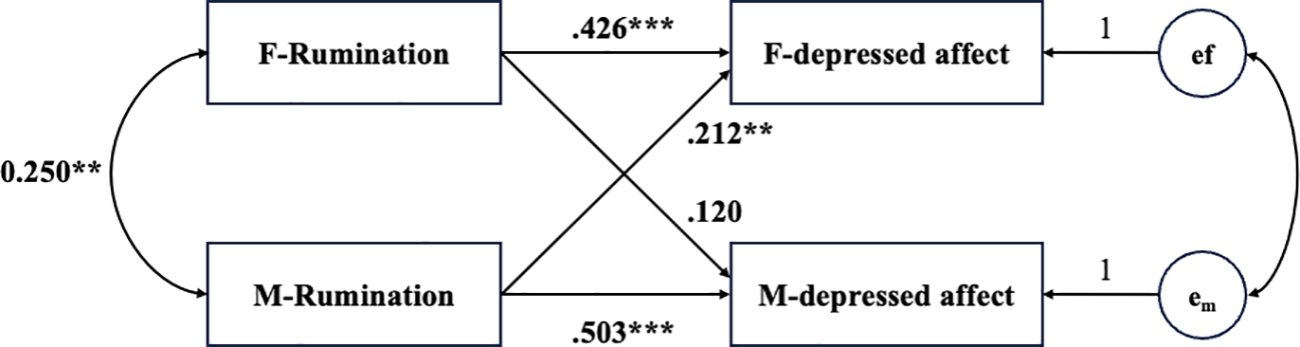
Actor–partner interdependence model predicting depressed affect from rumination. e_f_ and e_m_ represent the error terms of depression of female and male. Participant age and relationship duration were controlled for in the model, but are not displayed in the figure for simplicity. **p <.01, ***p <.001.

For the model with couples’ depressed affect as the outcome variables, the model fit the data well, χ²(2) = 1.09, *p* = .58, CFI = 1.00, TLI = 1.00, RMSEA = .001. Regarding the actor effect (see **Figure 1**), the results indicated that both males’ and females’ rumination were positively associated with their own depressed affect (females: *β* = .426, *p* <.001; males: *β* = .503, *p* <.001). For the partner effect (see **Figure 1**), higher levels of rumination in males were positively correlated with higher levels of depressed affect in their female partners (*β* = .212, *p* = .001), while females’ rumination was not significantly associated with their male partners’ depressed affect (*β* = .120, *p* = .100). Additionally, for the covariate effects, relationship duration was negatively associated with males’ depressed affect, but it was not correlated with females’ depressed affect. Participant age did not correlate with either males’ or females’ depressed affect.

**Figure 2:**
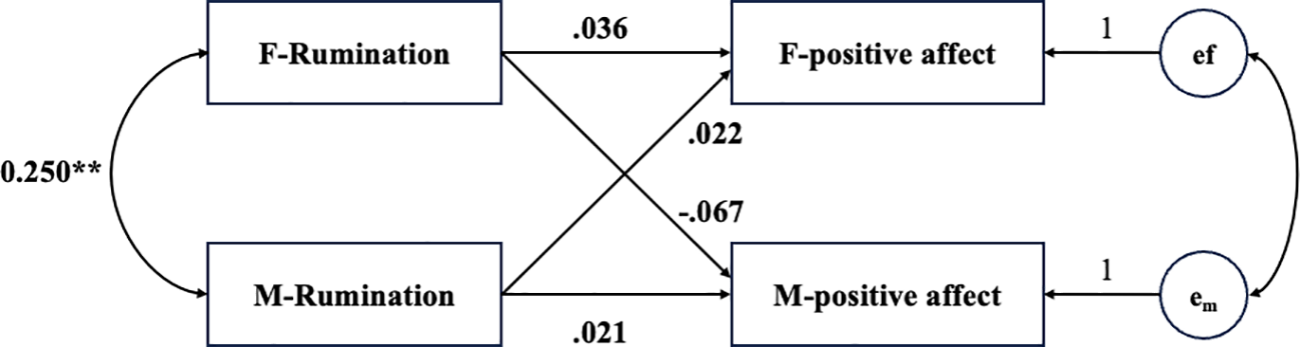
Actor–partner interdependence model predicting positive affect from rumination. e_f_ and e_m_ represent the error terms of positive affect of female and male. Participant age and relationship duration were controlled for in the model, but are not displayed in the figure for simplicity.

For the model with couples’ positive affect as the outcome variables, the model fit the data well, χ²(2) = 1.93, *p* = .91, CFI = 1.00, TLI = 1.00, RMSEA = .001. As shown in **Figure 2**, the results showed that neither males’ nor females’ rumination was significantly associated with their own positive affect, nor was it correlated with their partner’s positive affect.

**Figure 3:**
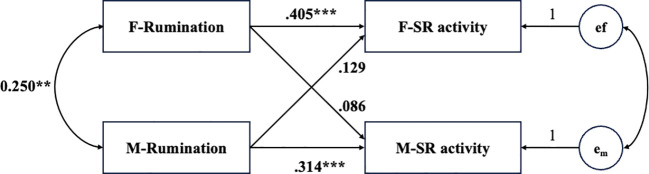
Actor–partner interdependence model predicting somatic and retarded activity from rumination. SR activity = somatic and retarded activity; e_f_ and e_m_ represent the error terms of somatic and retarded activity of female and male. Participant age and relationship duration were controlled for in the model, but are not displayed in the figure for simplicity. ***p <.001.

For the model with couples’ somatic and retarded activity as the outcome variables, the model fit the data well, χ²(2) = 0.48, *p* = .79, CFI = 1.00, TLI = 1.00, RMSEA = .001. Regarding the actor effect, the results indicated that both males’ and females’ rumination were positively associated with their own somatic and retarded activities (females: *β* = .405, *p* <.001; males: *β* = .314, *p* <.001). However, for the partner effect, neither males’ nor females’ rumination was significantly associated with their partners’ somatic and retarded activities (see **Figure 3**).

**Figure 4:**
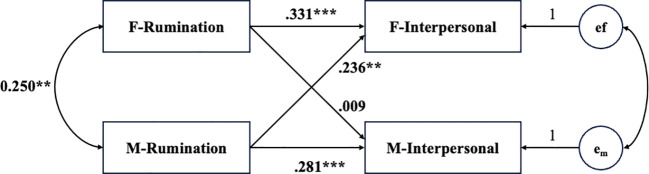
Actor–partner interdependence model predicting interpersonal distress from rumination. Interpersonal = interpersonal distress; e_f_ and e_m_ represent the error terms of interpersonal distress of female and male. Participant age and relationship duration were controlled for in the model, but are not displayed in the figure for simplicity. **p <.01, ***p <.001.

For the model with couples’ interpersonal distress as the outcome variable, the model fit the data well, χ²(2) = 2.62, *p* = .27, CFI = 0.989, TLI = 0.940, RMSEA = .045. As shown in **Figure 4**, regarding the actor effect, the results indicated that both males’ and females’ rumination were positively associated with their own interpersonal distress (females: *β* = .029, *p* <.001; males: *β* = .021, *p* <.001). For the partner effect, higher levels of rumination in males were positively correlated with higher levels of interpersonal distress in their female partners (*β* = .020, *p* = .001), while females’ rumination was not significantly associated with their male partners’ interpersonal distress (*β* = .001, *p* = .910). Additionally, for the covariate effects, relationship duration was negatively associated with both males’ and females’ interpersonal distress (females: *β* = -.007, *p* = .026; males: *β* = -.009, *p* = .003). depressed affect. However, participant age did not correlate with either males’ or females’ interpersonal distress.

## 4 Discussion

Previous studies investigating the influence of rumination on depressive symptoms have often focused on its impact on the onset and duration of these symptoms (37, 38), leaving its role in more nuanced aspects of depressive symptoms unclear. Moreover, most literature has examined the relationship between rumination and depressive symptoms at the individual level (1, 2), with few studies exploring this relationship on both intrapersonal and interpersonal levels. Additionally, little research has investigated potential gender differences in these associations, particularly among individuals in romantic relationships.

The present study expands current knowledge by examining the effects of rumination on four aspects of depressive symptoms (i.e., depressed affect, positive affect, somatic and retarded activity, and interpersonal distress) in Chinese young couples, as well as the influence of gender on these associations. Results indicated that individuals who reported higher levels of rumination tended to experience greater depressed affect, somatic and retarded activity, and interpersonal distress. Interestingly, gender differences emerged in how an individual’s rumination was associated with their partner’s depressive symptoms. Specifically, males who reported more ruminative thoughts had female partners who reported higher levels of depressed affect and interpersonal distress. However, the reverse was not true; females’ rumination was not associated with their male partners’ depressed affect or interpersonal distress. These findings deepen our understanding of how gender differences influence individuals’ psychological responses to their partners’ rumination, highlighting the need for more targeted support programs for females in romantic relationships.

We found actor effects for both males and females, as well as partner effects for females, in the domains of depressed affect and interpersonal distress. The actor effects align with response styles theory (8), which posits that rumination can exacerbate depressive symptoms (39). Regarding partner effects, limited research has compared the impact of rumination on depressive affect and interpersonal distress across genders. Our findings reveal that males who engage in more ruminative thinking tend to have female partners with higher levels of depressed affect and interpersonal distress. In contrast, females’ rumination did not correspond to increased depressed affect or interpersonal distress in their male partners.

Several explanations may account for this gender difference. First, socialization often encourages females to be more emotionally expressive and introspective (23, 24). This increased focus on emotions can heighten their vulnerability to their partner’s rumination, making them more likely to experience depressive symptoms in response (7). Additionally, females may feel a stronger sense of responsibility for the emotional dynamics in their relationships (25, 26). This relational sensitivity can lead them to be more vigilant about signs of conflict or distress, making their partner’s rumination more impactful on their perceptions of relationship issues and on their own depressive symptoms. However, it is important to note that these potential explanations were not directly examined in our study, underscoring the need for future research to explore them further.

Surprisingly, rumination was not associated with positive affect at either the intrapersonal or interpersonal level. Our findings align with Tumminia and colleagues’ (2020) study, which found that rumination was significantly associated with adolescents’ negative affect but not with their positive affect. It appears that rumination is more closely linked to negative feelings, such as depressed affect, within couples than to positive emotions. These findings support previous observations that simply eliminating sources of suffering, such as rumination, may not be enough to enhance well-being (40). The absence of rumination does not necessarily lead to increased positive affect for individuals or their partners.

Regarding somatic and retarded depression-related activities, our results revealed only actor effects, meaning that an individual’s own rumination influenced their own somatic and retarded activity, while no partner effect was observed. This actor effect is consistent with existing research that demonstrates a strong link between rumination and various negative somatic consequences (14). The perseverative cognition hypothesis suggests that rumination can initiate and sustain stress, leading to adverse physiological outcomes, such as disrupted eating and sleep patterns (41). Rumination appears to keep individuals focused on negative thoughts, preventing them from engaging in restorative activities or seeking effective solutions under stress.

Interestingly, no partner effect was found, meaning that one partner’s rumination did not seem to influence the other partner’s somatic and retarded activity. This may be due to the more internalized and personal nature of somatic symptoms, which are typically experienced and managed on an individual level (42). Unlike emotional or interpersonal distress, which may be more shared or observable between partners, somatic symptoms related to rumination—such as sleep disruption or changes in appetite—are likely less directly influenced by a partner’s behavior. These symptoms may primarily be driven by the individual’s own coping mechanisms and physiological responses, rather than external factors such as their partner’s ruminative response style.

### 4.1 Limitations

Our study was among the firsts to explore the relationship between rumination and depressive symptoms at both the intrapersonal and interpersonal levels in a heterogeneous sample of young couples from the community. These findings contribute to a deeper understanding of how rumination is linked to depressive symptoms in romantic relationships, while also highlighting the differential role of gender in these associations. However, there are some limitations to consider. First, the cross-sectional design of the study does not allow us to establish causality or test the direction of effects. Second, the study relied solely on self-reported data for all variables, which may introduce biases such as social desirability or inaccurate self-assessment. Despite these limitations, this study provides one of the firsts evidence of a partner effect of rumination on females’ depressive symptoms in romantic relationships. These findings underscore the importance of developing targeted psychoeducation programs for females, particularly those with male partners who demonstrate higher levels of rumination, in order to help prevent the future development of depression.

## Data Availability

The raw data supporting the conclusions of this article will be made available by the authors, without undue reservation.
